# Effects of low-GI biscuits as pre-loads or mid-meal snacks on post-prandial glycemic excursions in women with recent gestational diabetes: A protocol for a randomized crossover trial and an extended tailored intervention

**DOI:** 10.3389/fnut.2023.1122102

**Published:** 2023-03-23

**Authors:** Chunrong Li, Yan Gao, Tongyong Luo, Shiji Qin, Xue Yao, Ye Wen, Xue Wang, Jing Zhang, Qiong Zhong, Hao Shi, Jing Liu

**Affiliations:** ^1^Chengdu Women's and Children's Central Hospital, School of Medicine, University of Electronic Science and Technology of China, Chengdu, Sichuan, China; ^2^Women Health Department, Chengdu Jintang District Maternal and Child Health Hospital, Chengdu, Sichuan, China; ^3^Sichuan Provincial Hospital for Women and Children, Affiliated Women and Children's Hospital of Chengdu Medical College, Chengdu, Sichuan, China; ^4^Healthcare Department, Chengdu Jinjiang District Maternal and Child Health Hospital, Chengdu, Sichuan, China; ^5^Chengdu Tianyi Cuisine Nutritional Food Co., Ltd., Chengdu, Sichuan, China; ^6^Healthcare Department, Chengdu Jinniu District Maternal and Child Health Hospital, Chengdu, Sichuan, China

**Keywords:** gestational diabetes, pre-load, post-prandial glycaemic response, continuous glucose monitoring, glycaemic index

## Abstract

**Background:**

Increased post-prandial glycemic excursions contribute to the development of diabetes and have been observed in women with recent gestational diabetes mellitus (GDM) and with normal glucose tolerance at post-partum. As a convenient meal replacement, low-GI biscuits are helpful for improving glycemic excursions in patients with type 2 diabetes. However, it is unknown whether low-GI biscuits as pre-loads or mid-meal snacks have a better effect in diminishing post-prandial glycemic excursions from the individual level in women with recent GDM. Therefore, the aim of this trial is to tailor a better dietary strategy utilizing low-GI biscuits (Fitmeal) to improve post-prandial glycemic excursions through within-subject comparison in such a population and observe the long-term effect of a tailored dietary approach in glycemic control.

**Methods:**

We have designed a two-phase trial including a randomized, crossover, non-blinded trial in the first phase, followed by a 4-week tailored intervention in the second phase. A total of 52 post-partum women with recent GDM will be allocated into four meal plans: (1) Fitmeal pre-load 30 min before standard lunch meal (P+L), (2) Fitmeal as a mid-meal snack 2 h before standard lunch meal (S+L), (3) isocaloric standard control with co-ingestion of Fitmeal and standard lunch meal (CL) at the same time, and (4) placebo control with 200 ml of water taken 30 min before standard lunch meal (W + L), on four consecutive days. Acute post-prandial glycemic response (PGR) measured by continuous glucose monitoring (CGM) will be compared among the four meals. In the second phase, all participants will receive a 4-week tailored intervention using Fitmeal as pre-loads or mid-meal snacks based on within-subject PGR results from the first phase. Glycemic metrics, dietary behaviors, and psychosocial factors (e.g., quality of life, self-efficacy, perceived stress, and depression) will be examined at baseline and end-point.

**Discussion:**

This trial is expected to optimize the use of low-GI biscuits as pre-loads or mid-meal snacks in improving individual post-prandial glycemic excursions among women with recent GDM. Furthermore, the findings of this study will provide novel information on how to deliver an effective dietary intervention at the individual level and guide future clinical practice of medical nutrition therapy for diabetes prevention.

**Trial registration number:**

Chinese clinical trial registry, ChiCTR2200060923.

## Introduction

Gestational diabetes mellitus (GDM) is rising in prevalence with maternal hyperglycemia currently affecting one in every six pregnancies worldwide (1) and 18.9% in China (2). Compared with their peers, women with a history of GDM have a 7- to 10-fold higher risk of developing type 2 diabetes (T2DM) in the years thereafter (3, 4) and a 2-fold higher risk of cardiovascular disease within the first decade after the index pregnancy (5). With the development of the continuous glucose monitoring (CGM) technique, several studies found that the glucose fluctuation and post-prandial glucose excursions were significantly higher in women diagnosed with GDM during pregnancy and with normal glucose tolerance by OGTT at post-partum follow-up, as compared to women with normal glucose metabolism during pregnancy (6, 7). Glucose fluctuations during post-prandial periods exhibited a more specific triggering effect on oxidative stress than chronic sustained hyperglycemia, promoting the development and progression of T2DM (8, 9). Thus, it is suggested that interventional trials in such a population should be initiated early after delivery, targeting not only HbA1c and mean glucose concentrations but also acute post-prandial glucose excursions (10).

Lifestyle interventions initiated within 3 years of GDM during pregnancy targeting lowering calorie intake and body weight have been reported to be effective in reducing the risk of post-partum diabetes as compared to controls (11), but recently, a large randomized clinical trial (*n* = 1,812) has reported inconsistent result that a 12-month lifestyle intervention did not prevent glycemic deterioration, including the development of diabetes, among South Asian women with recent GDM within 2 years of childbirth (12). The low glycemic index (GI) diet is recommended by national guidelines to improve post-prandial glucose levels for individuals with diabetes or GDM (13, 14). However, in post-partum women with recent GDM, 6-month interventions with a low-GI diet alone or integrated with intensive lifestyle management did not halt or reverse glycemic deterioration although the risk of developing diabetes was reduced in the intervention group compared with usual care (15, 16). The limited success of current interventions might be explained by several reasons. First, increasing studies have shown that differences in individual post-prandial glycemic responses occur even when consuming the same food (17–19), but current lifestyle or dietary interventions are mainly based on one-size-fits-all advice and do not consider such differences between individuals. Moreover, if participants are unable to get immediate feedback on whether dietary approaches are effective in improving post-prandial glucose levels, it is hard for them to comply with recommended advice (20). Finally, post-partum women with recent GDM are commonly confronted with some barriers including lack of time and energy, limited childcare and social supports, emotional stress, workload-related stress of post-partum returning to work, insufficient knowledge or comprehension about GDM, and body image concerns (21, 22) such as competing demands may have a great impact on mothers' participation in dietary interventions. Thus, for post-partum women with recent GDM, whether a dietary intervention could improve post-prandial glucose immediately in a convenient and feasible strategy is a big challenge for increasing compliance with a recommended meal plan and then achieving long-term benefits.

Meal replacement is helpful for people with diabetes for being convenient and providing known level of calories and nutrients which facilitate meal planning and adherence to dietary recommendations (23), because of it being convenient and being able to provide known calorie amounts with specific macronutrient and micronutrient levels. In addition to replacing main meals and mid-meal snacks, meal replacement has been used as pre-load food given 15–30 min before a main meal, which is helpful to diminish post-prandial glycemic excursions, representing a novel dietary approach to control blood glucose (24–26). Recent studies reported that low-GI nutritional shakes pre-load given 30 min before each of three main meals reduced post-prandial 2 h blood glucose in patients with T2DM (26) and women with GDM (27). Our earlier study suggested that low-GI nutritional shakes given 20 min before each main meal significantly reduced post-prandial 2 h blood glucose and improved metabolic measurements in patients with obesity and metabolic syndrome in a 3-week before–after intervention study (28). As another form of meal replacement, low-GI biscuits with high protein and high fiber are more suitable to be applied as pre-load food compared with shakes using that biscuit is palatable, easy to carry, and can be consumed immediately without any pre-treatment as a mid-meal snack or as a part of main meal replacement. Several studies have shown that using low-GI biscuits as mid-meal snacks improved immediate post-prandial glucose levels (29), reduced post-prandial glucose levels 90 min after dinner (30), and improved satiety (31) in healthy subjects and individuals with T2DM. However, we are unaware of studies that have compared the benefits of using low-GI biscuits as pre-load foods and mid-meal snacks on the post-prandial glycemic response of the following main meal in a within-subject design, particularly in post-partum women with recent GDM.

A dietary strategy confirmed to be effective for reducing PGR individually is of paramount importance in increasing compliance with a recommended meal plan. Therefore, to optimize the use of low-GI biscuits in reducing individual PGR and provide valuable information in clinical practice of preventing T2DM for post-partum women with recent GDM, this proposed study will be conducted in two phases. The first phase (week 1) is designed with a randomized crossover trial with the main objective to compare the effect of low-GI biscuits consumed as pre-loads or as mid-meal snacks on individual PGR of the following main meal assessed by CGM. In the second phase (weeks 2–5), individualized intervention either with pre-loads or mid-meal snacks will be conducted depending on the results of personal PGR from the first phase. The objectives of the second phase are to observe before–after differences among all participants in their glycemic control using CGM metrics and glycated albumin (GA), dietary behavior, and psychosocial factors and compare those differences between the two dietary strategies. This article presents the development of the study design and procedure.

## Methods

### Study design

**Figure 1:**
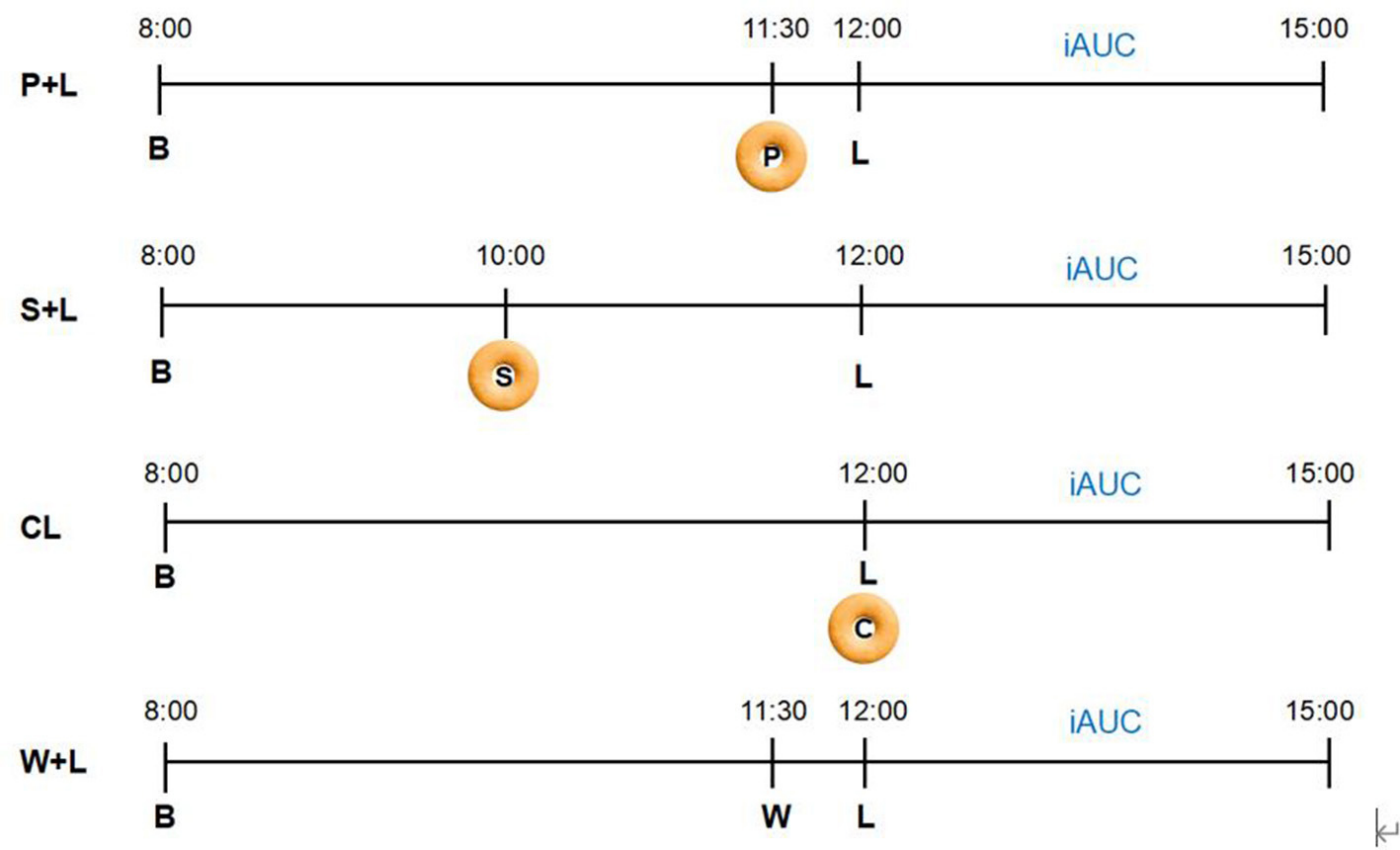
Randomized crossover design. Participants will complete four test meals over 4 consecutive days in a randomized counterbalanced order using Latin Square Design. Participants will be randomized to receive one of the four meal arrangement orders as follows: (1) P + L → S + L → W + L → CL; (2) S + L → CL → P + L → W + L; (3) CL → W + L → S + L → P + L; (4) W + L → P + L → CL → S + L. To diminish the glycemic impact from the last meal, breakfast, lunch, pre-load, and mid-meal snack foods are provided, with breakfast and lunch identical for 4 test days. 3h-iAUC after each lunch using continuous glucose monitoring (CGM) will be calculated as the primary outcome (P, Pre-load; S, Mid-meal snack; B, Breakfast; L, Lunch; W, Water; C, Co-ingestion; iAUC, Incremental areas under the curve of post-prandial glycemic response; P + L, Fitmeal pre-load 30 min before lunch; S + L, Fitmeal as mid-meal snack 2 h before lunch; CL, Co-ingestion of Fitmeal with lunch; W + L, Drinking water 30 min before lunch).

This is a randomized, crossover, and a non-blinded trial comparing the effects of four different test meals over four consecutive days on PGRs in women with recent GDM. The four meal plans are as follows: (1) Fitmeal pre-load 30 min before standard lunch meal (P + L), (2) Fitmeal as mid-meal snack 2 h before standard lunch meal (S + L), (3) co-ingestion of Fitmeal with standard lunch meal (CL) as isocaloric standard control, and (4) 200 ml of water taken 30 min before standard lunch meal (W + L) as a placebo control. All participants will complete four test meals during the trial period in a randomized counterbalanced order by Latin Square Design (Figure 1). Crossover randomization process is performed by the study biostatistician. Different orders for four test meals will be concealed in envelopes. Each subject is randomized by dispensing the next sequentially numbered subject code and opening the corresponding code envelope indicating the order for four test meals.

Moreover, a before–after intervention of 4-week duration based on within-subject meal test results will be followed for all participants to compare changes in PGRs from the baseline. The study will be carried out at four maternal and child healthcare hospitals in Chengdu. The protocol (CWCCH 2021027) has been approved by the ethics committee of Chengdu Women's and Children's Central Hospital, and written informed consent has been obtained from all participants. The study is conducted in accordance with the principles expressed in the Declaration of Helsinki.

### The study population

Women will be invited to participate in this study if they meet the following inclusion criteria: (1) those who are aged between 20 and 49 years; (2) those diagnosed with GDM during the latest pregnancy and <1 year post-partum at the beginning of the study; (3) those who have telephone access. Exclusion criteria are as follows: (1) those who are currently pregnant; (2) those who are still breastfeeding; (3) those who have been diagnosed with diabetes by OGTT test at post-partum follow-up or screening visit; (4) those who have been taking medicines that influence glucose metabolism; (5) those with severe heart, liver, and kidney dysfunction, psychiatric disorders, or other serious diseases that could reduce the ability to participate in the study; and (6) those who are unwilling to give informed consent.

### Recruitment

The recruitment of potential participants will occur by reviewing electronic medical records of women who delivered babies during the past 1 year at the research site by trained research nurses. Potential participants who meet the inclusion criteria will then be approached by a trained research nurse through a phone call to further confirm eligibility and provide study documentation in advance. Women who wish to participate will be invited to attend the screening visit and fasting for at least 12 h overnight before the screening visit will be required.

### Screening visit

The detailed medical eligibility questionnaire based on the abovementioned inclusion/exclusion criteria will be confirmed, and informed consent will be obtained at the screening visit. As part of the eligibility assessment, all potential participants will have OGTT testing and HbA1c to make sure the potential subjects do not have T2DM. If a woman has newly diagnosed T2DM (fasting glucose ≥7.0 mmol/l or 2-h glucose ≥11.1 mmol/l or HbA1c ≥6.5%), she will be excluded from this study and referred to a diabetes specialist for further T2DM diagnosis and treatment. If eligible, subjects will complete a self-administered questionnaire and undergo a physical examination at the same time, and continuous glucose monitoring devices will be fitted to each participant.

### Intervention

#### Low-GI Fitmeal™ biscuits (Fitmeal) and test meal

Fitmeal consists of soybean protein, whey protein, skimmed milk powder, whole egg, wheat powder, apple powder, stachyose, and L-arabinose. Each serving of Fitmeal (15 g) contains 7.6 g of protein, 1.8 g of fat, 1.9 g of fiber, and 5.2 g of carbohydrates, which provide 70 kCal of energy (Table 1, Tianyi Cuisine, Chengdu, China). GI value of low-GI biscuit (Fitmeal) is 43, according to test results by Peking Union Medical College Hospital and China National Research Institute of Food and Fermentation Industries following the ISO guidelines. The effect of Fitmeal on weight loss and blood glucose improvement has been described in a previous study (28). Standard test meals for breakfast and lunch for 4 consecutive test days will be provided to participants, and the composition and nutrient content of test meals are presented in Table 2.

**Table 1:** Nutrient composition of low-GI biscuits.

**Nutrient content**	**Low-GI biscuits (Fitmeal**™**)**		
	**100 g**	**15 g (2 biscuits)**	**22.5 g (3 biscuits)**
Energy (KJ)	1,929	289	434
Protein (g)	28	4.2	6.3
Fat (g)	20	3.0	4.5
Carbohydrate (g)	36	5.4	8.1
Fiber (g)	12.9	1.9	2.9
Sodium (mg)	556	83	125

**Table 2:** Composition and nutrient content of test meals.

**Test meal**	**W + L**	**P + L**	**CL**	**S + L**	**Breakfast**
Whole wheat bread (g)	70	70	70	70	35
Vegetable (g)	200	200	200	200	200
Egg (g)	50	50	50	50	50
Chicken breast (g)	50	50	50	50	50
Fitmeal (g)	–	22.5	22.5	22.5	–
Carbohydrate (g)	38.1	46.2	46.2	46.2	23
Protein (g)	28.6	34.9	34.9	34.9	24.3
Fat (g)	8.1	12.6	12.6	12.6	6.86
Fiber (g)	5.7	8.6	8.6	8.6	5.7
Energy (KJ)	1,404	1,838	1,838	1,838	1,032

W + L, Water + Lunch; P + L, Fitmeal pre-load + Lunch; CL, Co-ingestion of Lunch with Fitmeal; S + L, Fitmeal as mid-meal snack + Lunch. Vegetables include 100 g of tomato and 100 g of cucumber.

### First week

Participants consumed the same test meals for 4 consecutive days according to a randomized crossover order: (1) Fitmeal pre-load 30 min before standard lunch meal (P + L), (2) Fitmeal as mid-meal snack 2 h before standard lunch meal (S + L), (3) co-ingestion of Fitmeal with standard lunch meal (CL) at the same time, containing 46.2 g of available carbohydrates where the Fitmeal contributes 8.1 g and standard meal provides 36.1 g, and (4) drinking water 30 min before standard lunch meal (W + L). To control the impact of different types of breakfast on the glycemic response of the next meal, the same breakfast will be provided to all participants on 4 test days. Participants are instructed to start breakfast, mid-meal snack, pre-load, and lunch at 8:00 a.m., 10:00 a.m., 11:30 a.m., and 12:00 p.m., respectively, during test days. Participants will be suggested to consume biscuits for 5 min and test meals for 10–15 min with 200 ml of water. During each test day, participants are required to have a 12-h fast, without strenuous exercise performed, and without alcohol consumed during the past 24 h before the breakfast meals. Extra foods and beverages are not permitted to consume from the time of starting breakfast to 3 h post-lunch during each test day, with specific reasons and benefits informed by the research nurse. In the event that participants consume extra food during the test period, they are required to record in the paper logbook to monitor compliance and report to the research nurse in time. Even if not reported, abnormal glucose excursions monitored by CGM will also provide objective information to the research team for evaluation.

Participants will wear CGM (FreeStyle Libre 1, FSL1, Abbott Diabetes Care, Inc., Alameda, CA) that measures interstitial glucose concentrations for the first week. On days 1 and 2 of CGM use, women are instructed to eat at their diet routine for monitoring baseline glucose profile. On days 3–6, women are instructed and reminded by the research nurse to consume the test foods provided according to their randomized order. On day 7, the CGM will be removed, and data from days 3 to 6 will be extracted for comparison of different test meals. From day 1 to 6, participants are required to record their food diary and the time to start each meal using the paper logbook, additional photos of each meal should be taken for a clear memory at later counseling.

### Counseling and 4 weeks of follow-up from week 2 to 5

After removing CGM, each participant will receive a 20-min face-to-face counseling from a trained healthcare provider in each research site, including the explanation of baseline CGM glucose patterns and a comparison of PGRs of four test meals in a visual and individualized manner. Dietary advice about healthy food choices will be given as is the routine recommendation in China Medical Nutrition Therapy Guideline for Diabetes (36): (1) an appropriate amount of fish, eggs, low-fat milk, lean meat, and reduction in fatty meat in the diet, (2) more fiber-rich food, such as whole grains, vegetables, and fruits, (3) avoidance of simple sugars and refined carbohydrates, and (4) appropriate energy intake. Each participant's current status of body weight, physical activity, and dietary behaviors collected at the baseline survey will be considered in providing dietary advice.

A dietary approach of pre-load (P + L) or mid-meal snack (S + L) with lower iAUC from test results will be recommended to follow during the next 4 weeks. Considering rush time in the morning before breakfast, pre-load or snacks will be suggested to consume before lunch and dinner. For participants who are suggested to follow S + L, three Fitmeal at each morning tea and afternoon tea are asked to consume, approximately at 10 a.m. and 4 p.m., 2 h before lunch and dinner. If suggested to follow P + L, participants are asked to consume three Fitmeal 30 min before lunch and dinner, ~11:30 a.m. and 5:30 p.m. Blood glucose meter and testing supplies will be provided to participants for self-monitoring of blood glucose (SMBG) with six times a day in a manner of paired pre-meal and 1 h-post-meal, at least 2 days per week.

**Figure 2:**
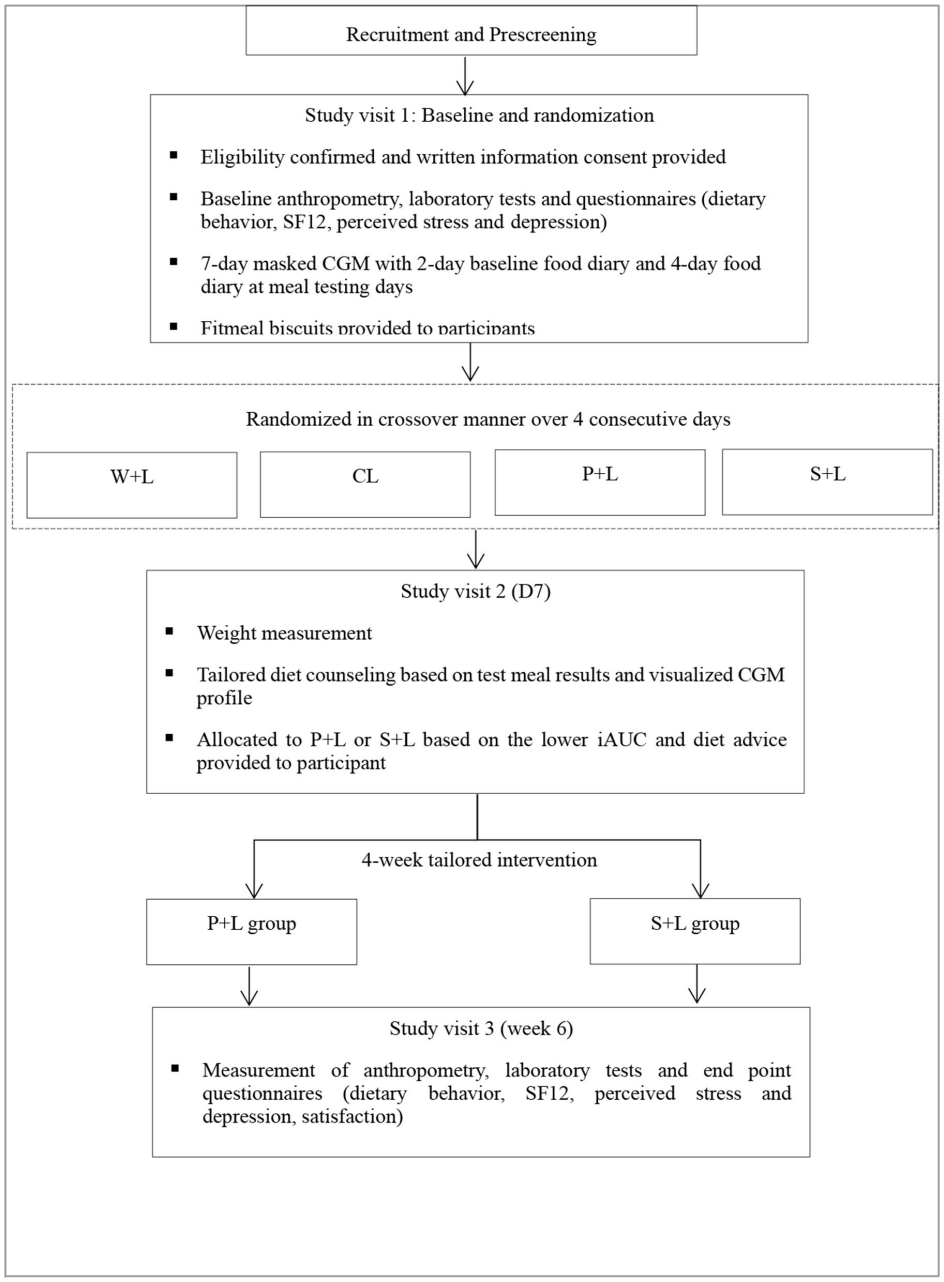
Study design flow chart.

From week 6, participants will be invited to the research site to complete an end-point questionnaire and physical examination, another CGM will be applied to monitor glucose profile for at least 72 h, and a food diary and the time to start each meal are required to record using the log book for 2 continuous days (midnight to midnight) from the second day of applying CGM. At the end of this trial, the comprehensive glucose profiles and the impact of diet on glucose levels will be reviewed and discussed with each participant in face-to-face counseling for facilitating their understanding and compliance with the dietary strategy to stabilize glucose levels. The study flow chart can be seen in Figure 2.

### On-site training for healthcare professionals and nurses

To make sure this proposed trial is implemented in each research site as a procedure, healthcare professionals and nurses working in the research sites will be recruited to take training provided by the research team. Their tasks are as follows: (1) to recruit and enroll the eligible participants; (2) to fit in CGM for participants and teach them how to use blood glucose meter for SMBG; (3) to set up automatic and individualized messages to remind each participant complying with each test meal and taking on-site measurements on schedule through an interactive WeChat Mini Program, in combination with telephone and WeChat for a double check; and (4) to provide dietary counseling and explaining CGM results to the participants. Moreover, dietitians and clinicians from the research team are available to provide advice and support about this trial implementation.

### Data collection

A baseline electronic questionnaire will be completed in a face-to-face interview with assistance from a research assistant at enrollment including sociodemographic data, medical history data, obstetric history, family history of diabetes, diet and physical activity behaviors during the last week, quality of life, stress, and depressive status. Sociodemographic, medical history data, and obstetric history of participants will be collected from the hospital medical records. In addition to the collection at enrollment, the 12-item Short Form Survey (SF-12) score (32) for the assessment of the quality of life, the Edinburgh Post-natal Depression Scale (EPDS) questionnaire (33) for the assessment of depressive symptoms, perceived stress (34), international physical activity questionnaire (IPAQ) (35) for evaluating physical activity level, and changes in diet behavior will also be completed at the moment of the end-point visit. Food diary and the time to start each meal will be required to record using a semi-structured paper logbook daily during the first week, at least 2 days from week 2 to week 5 paired with SMBG, and 2 consecutive days from the second day of CGM use at week 6. The electronic questionnaire at the end-point includes also questions concerning satisfaction with the intervention accepted.

Physical measurements (including weight, height, waist circumference, and body fat %) will be assessed at the enrollment and end-point visit. Participants will be measured barefoot and in light clothing. Weight will be recorded to the nearest 0.1 kg using a digital scale (HW-900B, He'Nan Lejia Electronic Technology co., Ltd, Zhengzhou). Height will be assessed to the nearest 0.1 cm with a calibrated wall-mounted stadiometer (HW-900B, He'Nan Lejia Electronic Technology Co., Ltd., Zhengzhou). Body mass index (BMI; in kg/m^2^) will be calculated as weight divided by square height. Waist circumference, defined as the midpoint between the lowest rib margin and the iliac crest, will be measured to the nearest 0.1 cm with a non-elastic tape. Measurements will be performed by a trained research nurse, following standard protocols.

Laboratory tests performed at baseline and end-point visits include fasting plasma glucose, 75 g 2-h oral glucose tolerance test (OGTT), glycated albumin (GA), triglycerides (TG), total cholesterol (TC), low-density lipoprotein (LDL), and high-density lipoprotein (HDL) cholesterol (Table 3).

**Table 3:** Data collection at evaluation of time points.

**Variables**	**Enrollment**	**Day 1**	**Day 2**	**Day 3**	**Day 4**	**Day 5**	**Day 6**	**Day 7**	**Week 2–5**	**Week 6**
**Questionnaire**										
Social-demographics	X									
Medical history	X									
Obstetric history	X									
Dietary behavior	X									X
IPAQ	X									X
Quality of life	X									X
Self-efficacy	X									X
Perceives stress	X									X
EPDS	X									X
Satisfaction										X
Food diary		X	X	X	X	X	X		X	X
**Laboratory tests**										
75 g OGTT	X									X
GA	X									X
Lipid profiles	X									X
**Glycemic measures**										
CGM measures	X	X	X	X	X	X	X			X
SMBG									X	
**Physical measures**										
Weight and BMI	X							X	X	X
Height	X									
Waist circumference	X									X
Body fat %	X									

Glycemic metrics will be calculated with values from CGM and SMBG. Participants who have signed the consent form will wear the CGM (FreeStyle Libre 1, FSL1, Abbott Diabetes Care, Inc., Alameda, CA) two times, monitoring for 7 days on week 1 and 3 days on week 6, respectively. The FSL1 is the most used biosensor for intermittent scanned CGM. All glucose data will be given each 15 min with a total of 96 readings every 24 h. From all these glucose measurements, several glucose metrics are calculated and reported on a 24-h basis average, including the percentage of time in a range from 3.9 to 10 mmol/L (TIR), percentage of time above range (TAR), percentage of time below range (TBR), a standard difference of mean glucose (SD), mean amplitude of glycemic excursions (MAGE), %CV, average glucose concentration (AG), incremental areas under the curve (iAUC), and post-prandial glycemic excursions (PE). Changes in glycemic metrics are calculated by subtracting the averaged values obtained on day 1 and 2 with CGM use at the baseline from the averaged values obtained on the second and third days of CGM use at the end-point. iAUCs will be calculated to represent the PGRs of four test meals from day 3 to 6, respectively. SMBG will be tested by glucose meter data (Qin Zhi, Sinocare, Inc., Changsha, China) six times a day in a paired test regimen at pre-meal and 1 h post-meal, at least 2 days per week from week 2 to week 5. SMBG frequency, average fasting, and pre-meal blood glucose concentrations, average 1 h post-prandial glucose concentrations (1hPG), and 1 h post-prandial excursions (1hPE) will be calculated. CGM and glucose meter data (An Wen/Qin Zhi, Sinocare, Inc. Changsha, China) will be uploaded automatically to the data platform and will be analyzed at the end of each monitoring period.

### Outcome

#### Primary outcome

The 3-h post-lunch incremental areas under the curve of post-prandial glycemic responses (iAUC) using CGM are compared between the low-GI biscuits as pre-load food (P + L) or mid-meal snack (S + L) and their isocaloric standard control of co-ingestion (CL) and placebo control (W + L) in women with recent GDM. Primary outcomes also include differences in CGM measures (AG, SD, %CV, MAGE, TIR, TAR, TBR, and PE) and GA after a 4-week intervention compared with those variables at the baseline.

#### Secondary outcomes

Secondary outcomes are differences in weight, glucose tolerance, dietary behaviors, quality of life, self-efficacy, perceived stress, and depression after a 4-week intervention compared with those variables at the baseline, and those differences will also be compared between two dietary strategies.

### Sample size

The sample size of the trial is estimated using PASS 15 Power Analysis and Sample Size software (NCSS, Kaysville, UT, United States). Assuming that the standard deviation (SD) is 32.9 mmol·min/L, a sample size of *n* = 39 provides 90% power to detect a change of 20 mmol·min/L in iAUC (*p* < 0.05) with Bonferroni correction, based on the previous study (36). Anticipating a 20% dropout rate, 49 subjects should be enrolled to obtain a final sample size of 39. Considering participants enrolled in four hospitals, 52 subjects will be enrolled with 13 subjects for each center.

### Statistical analysis

The incremental areas under the curve (iAUC) will be calculated by using the trapezoidal rule (37) for PGR. All areas below pre-prandial glucose are excluded from the calculations. Two-factor repeated-measures analysis of variance (ANOVA) is performed to analyze the effects of treatment × time on outcome variables measured over the crossover study period, including iAUC for PGR and post-prandial glucose excursion. When treatment and time interaction are statistically significant, one-factor ANOVA and Bonferroni's *post-hoc* tests for multiple comparisons are performed to investigate the effect of treatment on the aforementioned measurement of PGR. Comparison between baseline and end-points is assessed by paired Student's *t*-test. Values are presented as mean ± SEM unless otherwise indicated. *P*-values of <0.05 are considered statistically significant. All analyses are performed with SPSS software version 25 (SPSS Inc., Chicago, IL, USA).

## Discussion

With a high risk to develop T2DM, women with a history of GDM should receive appropriate intervention early after delivery (38). A clinically feasible intervention targeting post-partum women with recent GDM should be convenient to follow and easy to experience effectiveness in the shortest possible time, which would facilitate long-term compliance of participants. To the best of our knowledge, this proposed study will be the first to optimize the use of low-GI biscuits as a different dietary approach to diminishing post-prandial glycemic excursions and examine the efficacy of a tailored dietary intervention for post-partum women with recent GDM.

Current evidence suggested that lifestyle interventions within 3 years of GDM pregnancy reduced the risk of post-partum diabetes compared to controls, but traditional interventions targeting dietary energy limitation and weight control are challenging in evaluating whether the glycemic control is effective (15, 38, 39), in addition to difficulties in maintaining long-term compliance (11). Recent studies evaluating the effectiveness of diabetes prevention for women with prior GDM through a 6-month intensive lifestyle intervention showed inconsistent results in glycemic control, with a significant reduction in FBG and 2hPG of OGTT observed in the intervention group compared with controls, but HbA1c in two groups showed a clear trend of increase, and there is no difference between the two groups after 6-month intervention (15, 40). These inconsistent results suggest that it could be ignored about acute glucose fluctuations during post-prandial periods, which have been observed to be higher in women with recent GDM and with normal glucose tolerance by OGTT at post-partum follow-up measured by CGM, as compared to women with normal glucose metabolism during pregnancy (6, 7). Furthermore, increased acute post-prandial glucose contributes to the development of diabetes and is widespread in the Chinese population with diabetes and prediabetes. According to a national survey in China, more than 70% of the participants with prediabetes had isolated impaired glucose tolerance (41). Thus, a dietary intervention strategy for post-partum women with recent GDM should target on diminishing post-prandial glycemic excursions, which are sensitive to dietary adjustment, and the effect can be monitored in real-time.

In addition to commonly used mid-meal snacks (13, 42), pre-load represents a novel dietary approach to lower post-prandial glycemic excursions (14, 43). A variety of foods, such as olive oil, milk proteins, fruits, and low-GI nutritional shakes, have been demonstrated to improve post-prandial glucose levels (26, 27, 44–47). However, previous studies mainly examined the influence of pre-load on acute PGRs only (44–47) or assessed changes in 2hPG after pre-load intervention for 8–12 weeks in healthy population, women diagnosed with GDM, or individuals with T2DM (26, 27). Due to individual glycemic response differences occurring even when consuming the same food (17), it is necessary to integrate individualized PGR into a specific dietary approach, i.e., pre-load or mid-meal snack, with a continuing period of intervention based on the optimal approach to confirm the effect of specific dietary approach on improving glycemic excursions in real life. Being more convenient to consume without any pre-treatment compared with low-GI nutritional shakes, low-GI biscuits are more suitable to be applied as pre-load food for post-partum women with recent GDM. Based on the literature and our preliminary data, the proposed intervention will compare the impact of low-GI biscuits as pre-load food or commonly used mid-meal snacks upon post-prandial glycemic excursions measured by CGM. Furthermore, the present trial is intended to optimize the use of low-GI biscuits as a different dietary approach in improving post-prandial glycemic excursions, in combination with continuing intervention to evaluate whether the optimal approach would promote dietary self-management behavior and improve related psychosocial factors for post-partum women with recent GDM.

This proposed trial has some limitations. First, the free-living conditions of participants may raise non-compliance and bias as composed with in-site tests, although standard main meals for breakfast and lunch will be provided to participants to test acute PGRs by CGM. We will pre-set the automatic messages through WeChat Mini-Program to remind participants complying with each test meal, and the time to start each test meal will be confirmed in time by trained nurse. Second, CGM will be applied only two times at the baseline and after intervention rather than the whole period of intervention. It may not be possible to analyze the comprehensive glucose profile changes during the intervention, but a finger-prick blood glucose monitor will be provided to take daily blood glucose tests as a recommended regimen. Third, there is no washout period in-between 2 test days to minimize any carryover effects, but the same standard breakfast is provided to each participant to diminish the impact. Finally, the second stage of intervention is pragmatic and tailored based on optimal results from the first stage, it is impossible to implement randomization or to blind participants and investigators to provide robust evidence for confirming which dietary approach is better in improving post-prandial glycemic excursions with a 4-week intervention.

To minimize potential bias due to the study limitations, this study uses several approaches to reduce potential bias. First, to reduce the in-between differences in research sites, four tertiary hospitals in Chengdu city are selected for recruiting participants. Healthcare professionals and nurses working in the research sites are recruited to take training provided by the research team. Second, to improve data collection quality and reduce information bias, all electronic questionnaires in the proposed trial will be collected in a face-to-face interview by trained research assistants who are blinded to the treatment order and group assignments. All samples for laboratory tests are collected for centralized testing. Third, the study biostatistician, involved in data handling and analysis, will be blinded to participant study treatment and any potential identifiers.

At present, there is a shortage of evidence providing clinical practice and dietary management modes for post-partum women with recent GDM in China. We propose an innovative and pragmatic study that aims to assess the effectiveness of different dietary strategies in improving post-prandial glucose excursions in women with recent GDM. The proposed study will be a randomized crossover design, followed by a 4-week tailored intervention based on an optimum strategy that may facilitate compliance and self-efficacy in dietary self-management. We hope that the implementation of this protocol would help to provide a feasible and effective dietary management approach and behavioral strategy for improving post-prandial glucose excursions in the shortest possible time and contribute to reduce the incidence of T2DM in post-partum women with recent GDM in the long term.

## Ethics statement

The studies involving human participants were reviewed and approved by Medical Ethics Committee of Sichuan Jinxin Women and Children Hospital. The patients/participants provided their written informed consent to participate in this study. Written informed consent was obtained from the individual(s) for the publication of any potentially identifiable images or data included in this article.

## Author contributions

CL: study conception and manuscript writing. YG: study conception and study coordination. TL: manuscript writing and data analysis. SQ: manuscript writing. XY, YW, QZ, HS, JZ, and XW: data collection. JL: study conception, manuscript writing, and study coordination. All authors contributed to the article and approved the submitted version.
